# Supplementary material for: NOTCH1 activation compensates BRCA1 deficiency and promotes triple-negative breast cancer formation
Source: Nat Commun. 2020 Jun 26;11:3256. doi: 10.1038/s41467-020-16936-9 (PMC7320176; doi:10.1038/s41467-020-16936-9)
Supplement: Supplementary file 1 — Supplementary Information [file 41467_2020_16936_MOESM1_ESM.pdf]

Supplementary Information

NOTCH1 Activation Compensates BRCA1 Deficiency and Promotes Triple-Negative Breast Cancer Formation

Miao et al.

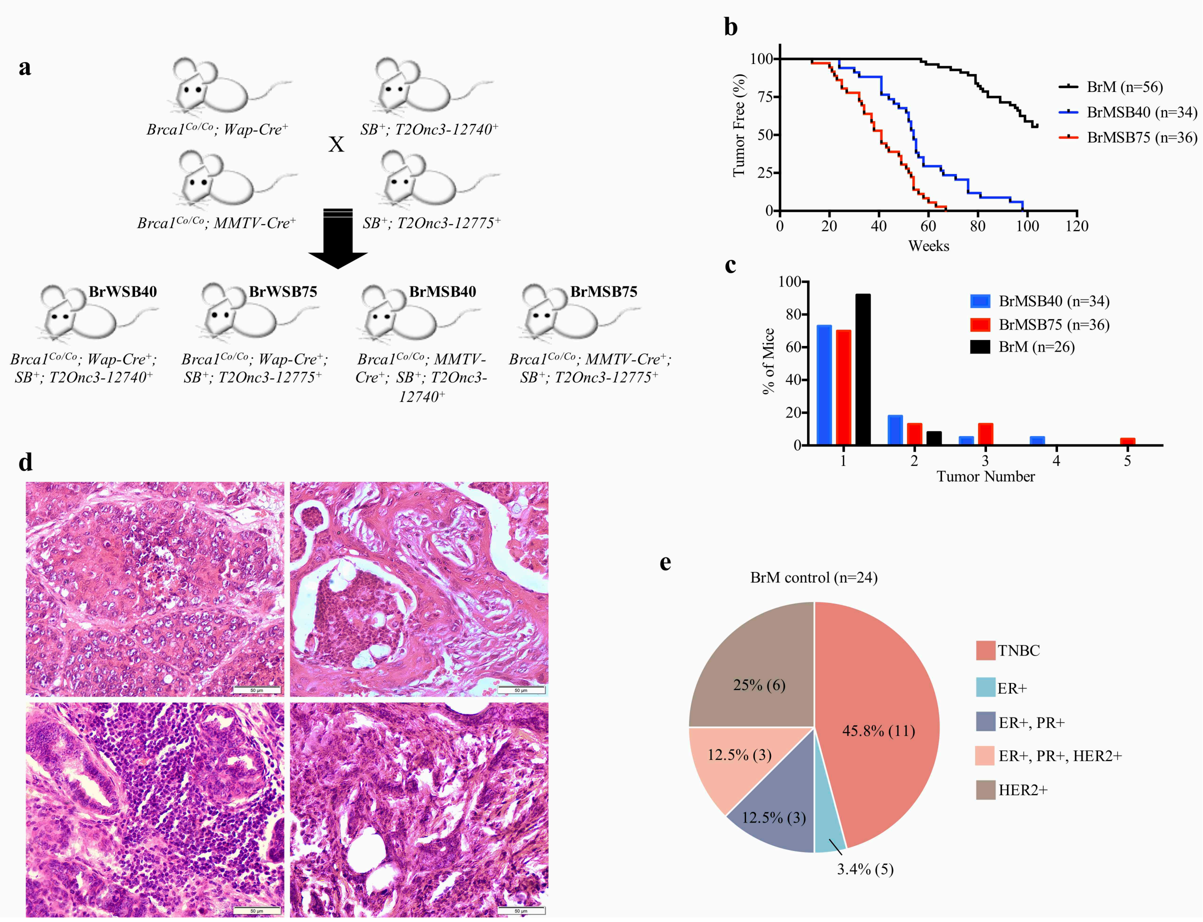

Supplementary Figure 1. SB insertional mutagenesis drives mammary tumourigenesis in female mice with mammary-gland-specific knockout of Brca1 (**a**) Workflow of mouse crosses. (**b**) Kaplan-Meier curve showing the mammary tumour-free rate for the indicated genotypes. BrMSB40 (n=34) and BrMSB75 (n=36) showed increased tumourigenesis compared to BrM control mice (n=56). BrMSB40 versus BrM (p<0.0001); BrMSB75 versus BrM (p<0.0001); by the log-rank test. (**c**) Numbers of tumours per mouse in different groups. (n=numbers of mice). (**d**) Histologic diversity of representative mammary tumours as revealed in H&E-stained sections n=30 for the BrWSB group, n=10 for the BrW group. (e) TNBC incidence in BrM group.

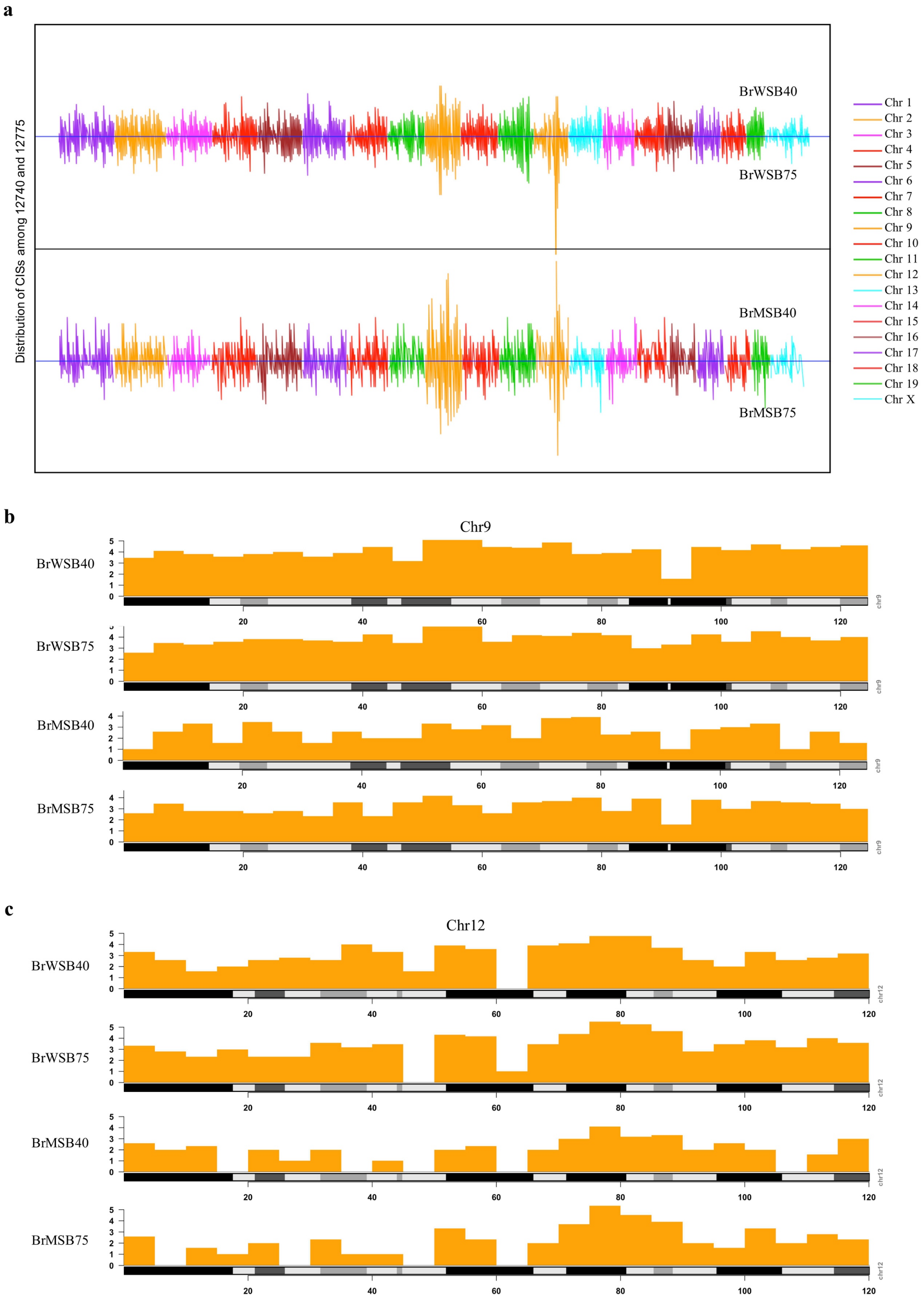

Supplementary Figure 2. Common insertion site analysis. (**a**) Distribution of all CISs identified in the mouse genome. A positive value indicates CISs identified from the 12740 strain, and a negative value represents CISs identified from the 12775 strain. The CISs did not show obvious distribution bias in chromosomes 9 (**b**) and 12 (**c**), which are the original sites of transposons in strains 12740 and 12775, respectively.

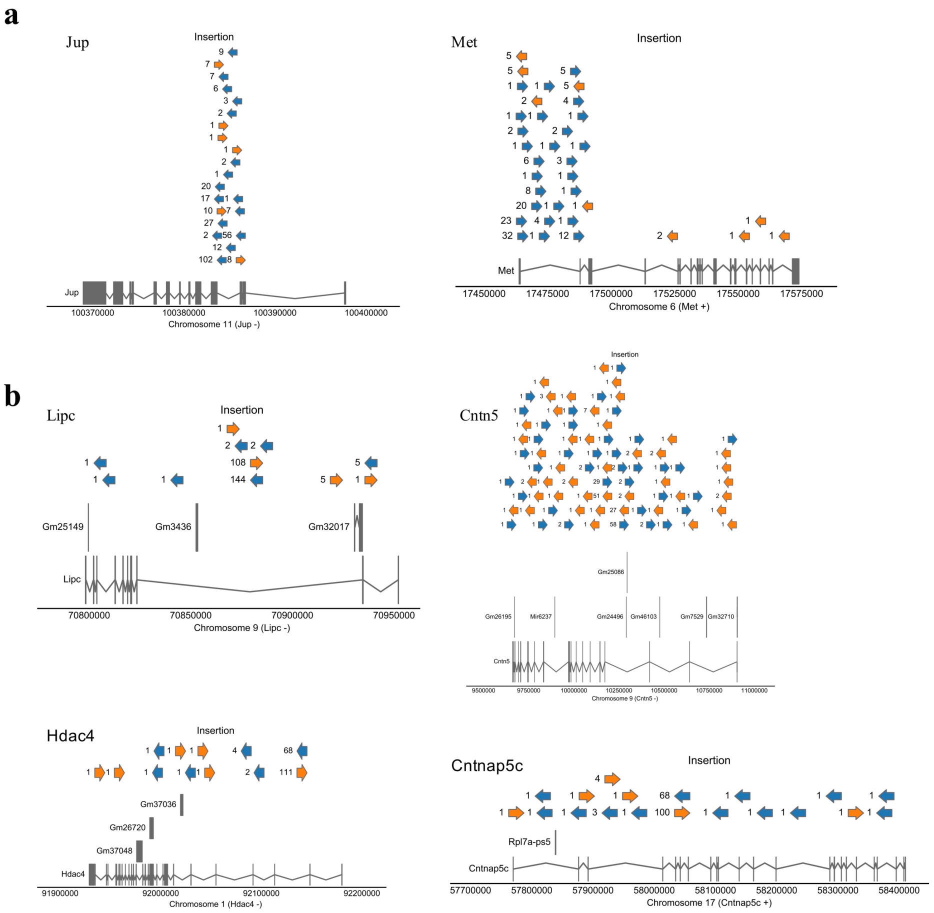

Supplementary Figure 3. Representation of the distribution of CISs as putative oncogenic drivers (**a**) and tumour suppressors (**b**).

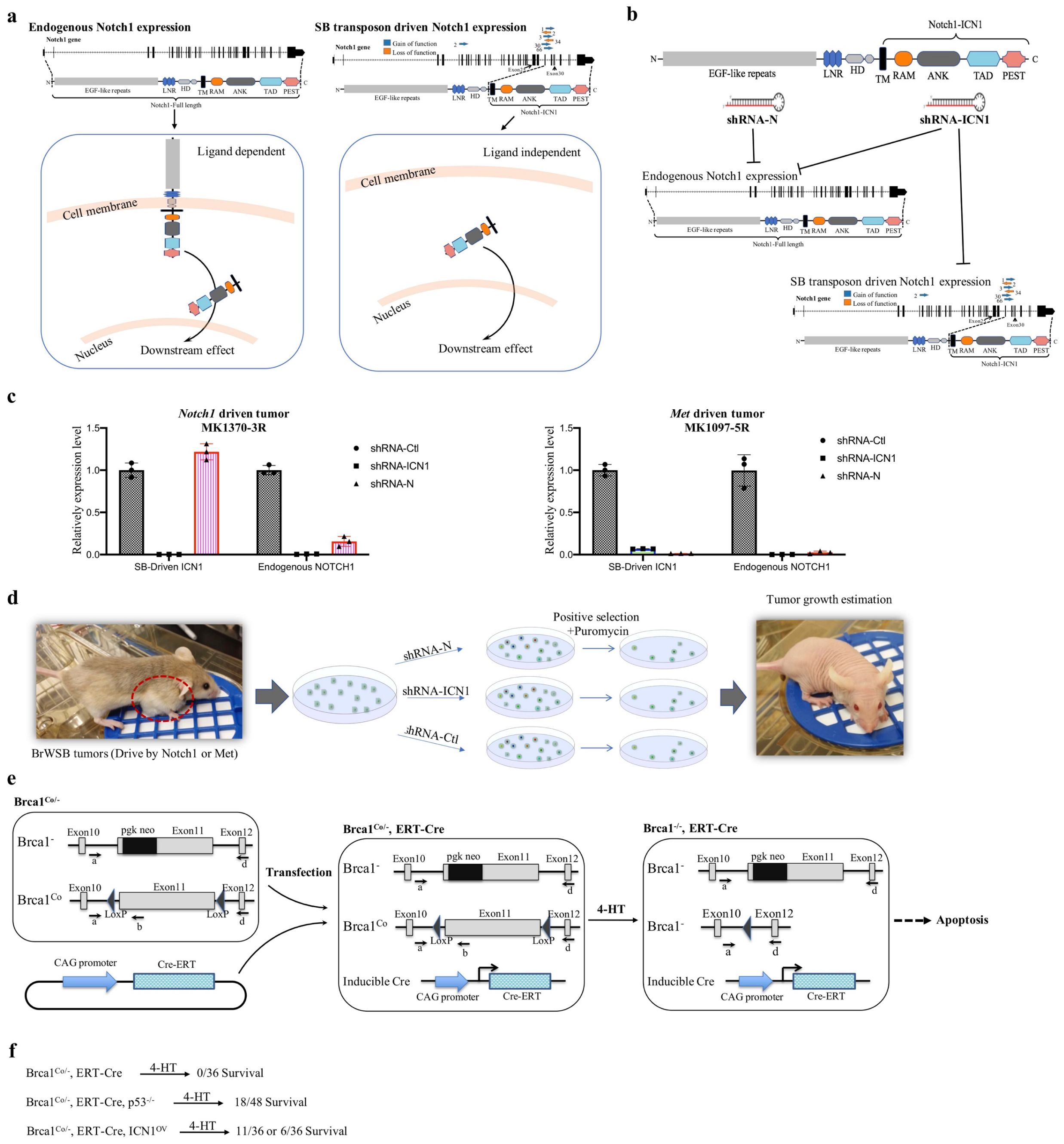

Supplementary Figure 4. In vivo validation of oncogenic function for Notch1. (**a**) Illustration of the endogenous Notch1 expression, which activation shows ligand dependent manner. And SB transposon driven Notch1 expression, which activation shows ligand independent manner. (**b**) shRNAs were designed against the N-terminus of endogenous Notch1 or SB transposon derived ICN1. (**c**) Q-PCR analysis of endogenous Notch1 and SB driven ICN1 in Notch1-driven tumours (MK1370-3R) and non-Notch1-driven tumours (MK1097-5R) after shRNA knockdown; n=3 biologically independent experiments. Data are presented as mean values ± s.d. (**d**) Tumours were collected from BrWSB mice and then infected with shRNA-lentivirus to knock down Notch1, followed by puromycin selection and inoculation of cells

into nude mice to assess tumour formation. (**e, f**) Conditional Brca1 mutant allele in Brca1Co/-;Cre-ERT2 ES cells. The cells contain a conditional knockout allele with exon 11 flanked by loxP sites and another Brca1 allele knocked out by a neomycin gene. Meanwhile, an inactive Cre-ERT-fused expression gene has been stably integrated into the cells. Upon treating the cell with 4-HT, the Cre enzyme will be transported into the nucleus to cut the Brca1 conditioned allele to generate Brca1-deficient cells. We generated lentiviral vectors carrying our candidate gene or sgRNA for Cas9-induced knockout. The viral vector is designed to contain a puromycin gene, which is resistant to puromycin selection. ES cells will be seeded into 6-well plates with the same cell number followed by 4-HT treatment to induce Brca1 knockout. Seven days later, 36 colonies were picked up for genotype to determine the Brca1 knockout genotype, then estimate the rescue effect of candidate genes.

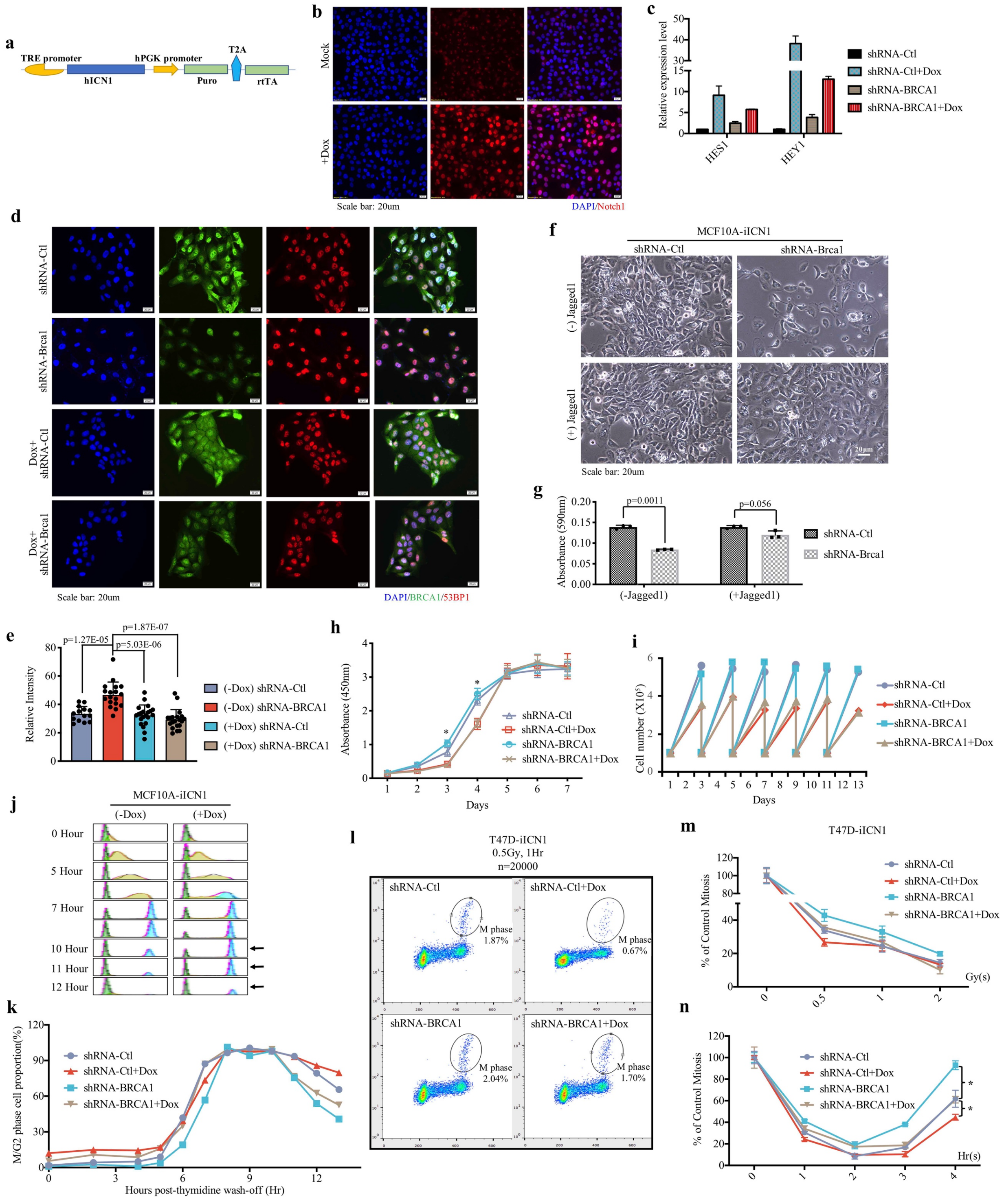

Supplementary Figure 5. Notch1 activation regulates cell cycle progression through the ATR-CHK1 axis. (**a**) Structure of the tet-on system used to overexpress ICN1. (**b**) Immunofluorescence staining indicating ICN1 overexpression after Dox induction. (**c**) quantitative analysis of Notch1 targeting gene expression level after Dox administration. (**d**) IF staining of 53BP1 to indicate DNA damage at 48 hours after BRCA1 acute knockdown with or without ICN1 overexpression. (**e**) Intensity quantification of 53BP1 to indicate DNA

damage at 48 hours after BRCA1 acute knockdown with or without ICN1 overexpression; n=14-21 independent cell measurements. (**f**) Activation of ICN1 by Jagged1 suppressed cell death caused by Brca1 acute knockdown in MCF10A cells. (**g**) Quantification analysis by MTT assays regarding the rescue effect of Notch1's ligand on BRCA1 deficiency. Analysis on the 3rd day after Dox and/or lentivirus with shRNA-BRCA1 induction; n=3 biologically independent experiments. (**h**) Growth rate of MCF10A cells under ICN1 overexpression and/or Brca1 knockdown. Cell number was measured daily by using CCK-8 assays. Four repeats were used to determine the SD. * indicates $p<0.05$. (**i**) Growth rate of MCF10A cells under ICN1 overexpression and/or Brca1 knockdown. Cell number was measured every three days, then re-plating the cells. The shRNA-BRCA1 adapted cells were used for this assay. Three repeats were used to determine the SD. (**j**) Cell cycle analysis of MCF10A cells after thymidine synchronization. Arrows indicate the difference after ICN1 overexpression at 10–12 hours. (**k**) Dynamic change in G2/M phase cell proportion at different time points after thymidine synchronization under ICN1 overexpression and/or Brca1 knockdown conditions. (**l**) Mitotic index analysis of T47D cells overexpressing ICN1 and/or with Brca1 knockdown. Cells were collected at 1 hour after 0.5 Gy irradiation treatment. Over 20,000 cells were counted and assayed in triplicate at each dosage or time point. (**m**) MI analysis of T47D cells Cells were treated with different doses of irradiation and measured at 1 hour; n=3 biologically independent experiments. (**n**) MI analysis of T47D cells collected at different time points after 0.5 Gy irradiation treatment; n=3 biologically independent experiments. Data are presented as mean values ± s.d. * indicates $p<0.05$.

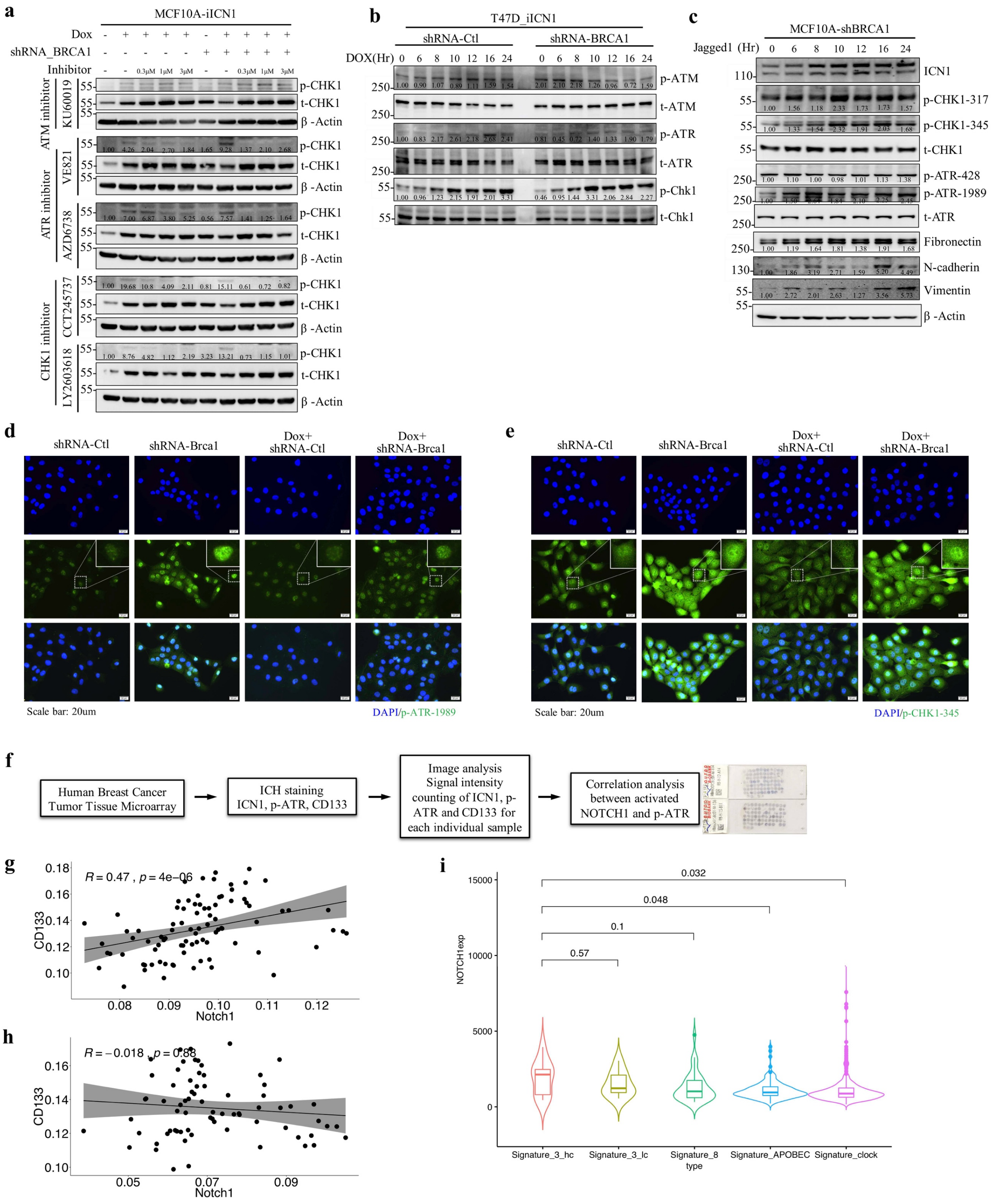

Supplementary Figure 6. Notch1 activation regulates ATR-CHK1 axis. (**a**) Western blot analysis of pCHK1 levels after ICN1 induction when treated with a CHK1 inhibitor (CCT245737, LY2603618), ATR inhibitor (VE821, AZD6738) or ATM inhibitor (KU60019). (**b**) Western blotting analysis of cell cycle checkpoint proteins at different time points after Dox administration in T47D cells. (**c**)Western blotting analysis of cell cycle checkpoint proteins and EMT marker proteins at different time points after Jagged1

administration in MCF10A cells. (**d**, **e**) IF staining of p-ATR and p-CHK1 at in BRCA1 knockdown or parental MCF10A cells after Dox administration. (**f**) Flowchart illustrating the workflow of human tissue microarray IHC staining. (**g**, **h**) Immunohistochemistry staining of Human TNBC patient tissue microarray for target proteins. The scatter plots indicate positive correlation among ICN1 and CD133, which is serve as a negative control (n=90 for g, n=72 for h). (**i**) NOTCH1 expression level analysis in different patient cohort to indicate NOTCH1 correlated with homologues recombination features. Kruskal–Wallis test was used to determine the significance of the difference between the different sets of data; two-sided test was used. Data are presented as mean values ± s.d. The violin shape indicates the distribution of individual values.

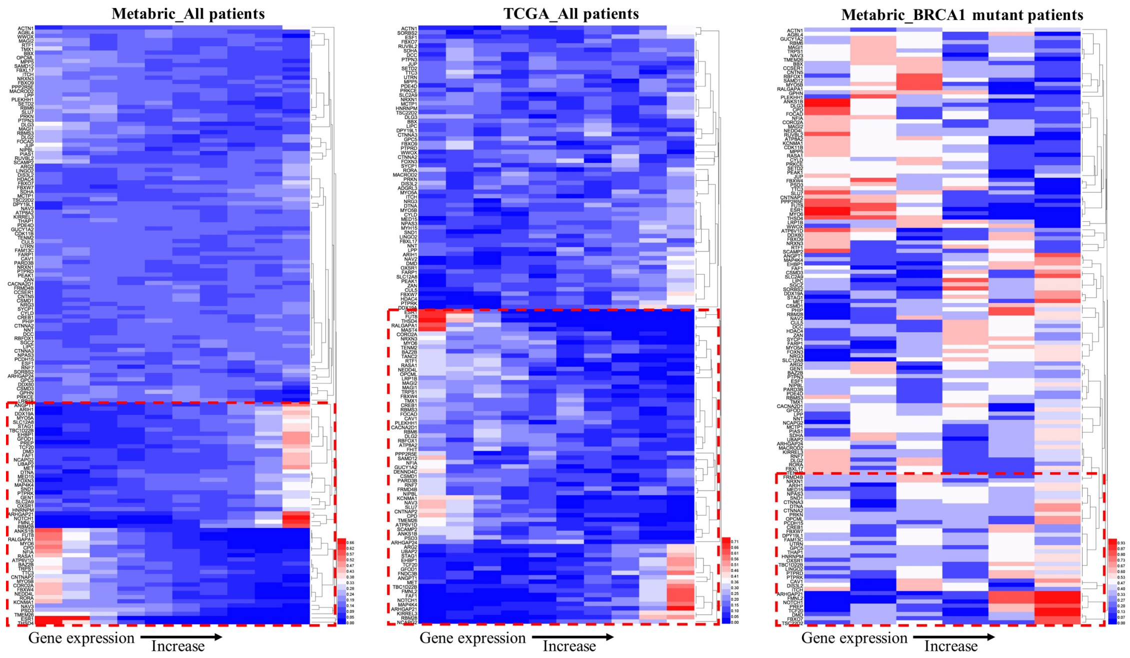

Supplementary Figure 7. Relationships between candidate gene expression levels and TNBC incidence rates in all breast cancer patients and BRCA1 mutant patients. Samples were separated into 10 or 6 cohorts based on the individual gene expression level, and the TNBC incidence rate for each single cohort was determined and is illustrated in the heatmap. Datasets are from TCGA and Metabric.

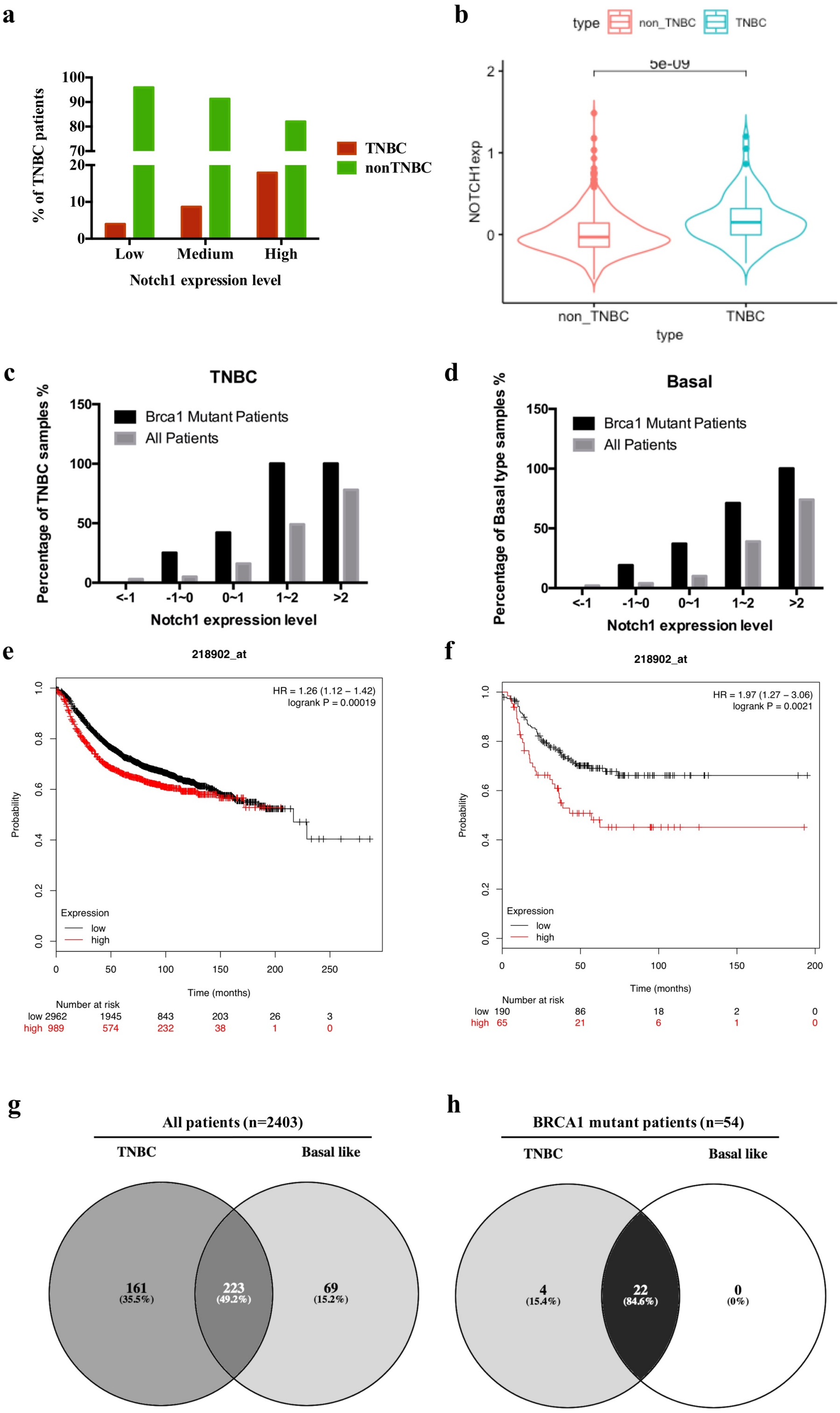

Supplementary Figure 8. Correlation analysis between Notch1 expression and TNBC incidence. (**a**, **b**) Correlation between Notch1 protein level and tumour subtype. Datasets are from TCPA. NOTCH1 protein expression levels are significantly higher in TNBC patients compared with nonTNBC patients (0.0019±0.24 vs. 0.18±0.29) Kruskal–Wallis test was used to determine the significance of the difference between the different sets of data; two-sided test was used. Data are presented as mean values ± s.d. The violin shape indicates the

distribution of individual values. (**c**) The relationship between Notch1 expression level and TNBC incidence rate in BRCA1 mutant patients or all patients. (**d**) Relationship between Notch1 expression level and basal type breast cancer incident rate in BRCA1 mutant or all patients. (**e**) Kaplan-Meier survival curve among NOTCH1 high- and low-expression patients. (**f**) Kaplan-Meier survival curve among NOTCH1 high- and low-expression TNBC patients. Correlation analysis between TNBC and the basal type of breast cancer in all patients (**g**) and BRCA1 mutant patients (**h**).

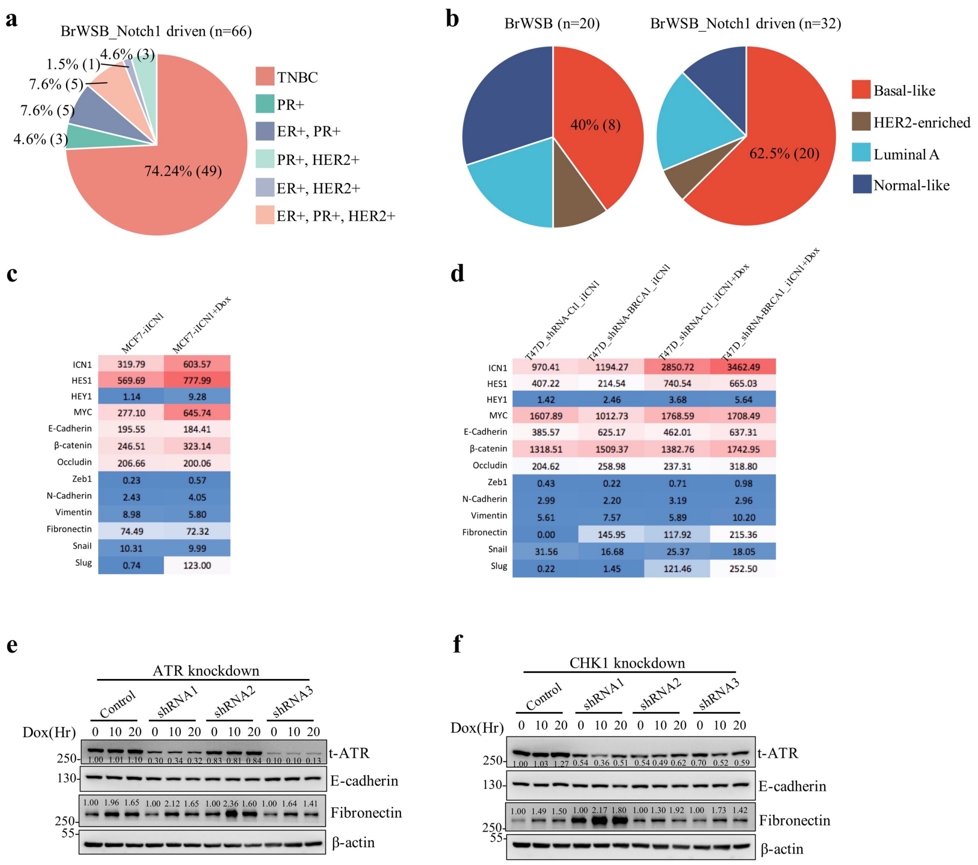

Supplementary Figure 9. Notch1 stimulates Brca1-related TNBC progression. (**a**) Statistical analysis of TNBC incidence in Notch1-driven SB tumours. (**b**) Statistical analysis of basal-like tumour incidence in Notch1-driven SB tumours. (**c**) Quantification PCR analysis of TNBC marker genes in MCF7 cells after ICN1 overexpression. (**d**) Quantification PCR analysis of TNBC marker genes in T47D cells after ICN1 overexpression with/without BRCA1 knockdown. (**e**) Western blotting analysis of expression of E-Cadherin and Fibronectin during a time course of ICN1 induction upon knockdown of ATR. (**f**) Western blotting analysis of expression of E-Cadherin and Fibronectin during a time course of ICN1 induction upon knockdown of CHK1.

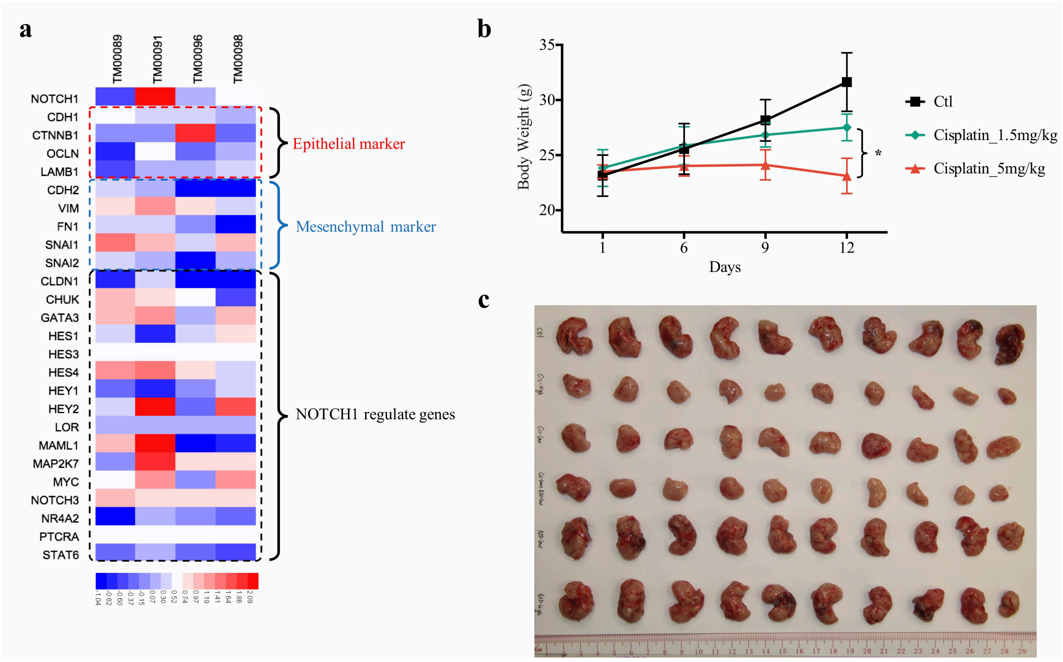

Supplementary Figure 10. In vivo combined drug treatment of TNBC tumours. (**a**) Comparison of gene expression between different PDX models. Genes circled with a dashed line are epithelial markers, genes circled with a blue dashed line are mesenchymal markers, genes circled with a black dashed line are regulated by activated NOTCH1. Based on the heatmap, model TM00091 shows the highest level of NOTCH1, as well as high levels of mesenchymal markers and NOTCH1 target genes. Therefore, we chose this PDX model for further in vivo drug treatment. (**b**) Body weight analysis of high-dosage and low-dosage of cisplatin treatment of the PDX model (TM00091). Six mice were used for each treatment group. * indicates p<0.05. Data are presented as mean values ± s.d. (**c**) Picture of TM00091 tumours from cisplatin single or combine treatment groups. Five mice were used for each treatment group.
