# Supplementary material for: NOTCH1 activation compensates BRCA1 deficiency and promotes triple-negative breast cancer formation
Source: Nat Commun. 2020 Jun 26;11:3256. doi: 10.1038/s41467-020-16936-9 (PMC7320176; doi:10.1038/s41467-020-16936-9)
Supplement: Supplementary file 3 — Description of Additional Supplementary Files [file 41467_2020_16936_MOESM3_ESM.pdf]

**Description of Additional Supplementary Files**

File Name: Supplementary Data 1
Description: Mouse Tumor collection summary

File Name: Supplementary Data 2
Description: Common insertion site list annotation

File Name: Supplementary Data 3
Description: Candidate genes of BrWSB group

File Name: Supplementary Data 4
Description: Candidate genes of BrMSB group

File Name: Supplementary Data 5
Description: Pathway enrichment analysis of candidate genes

File Name: Supplementary Data 6
Description: GO enrichment analysis of candidate genes

File Name: Supplementary Data 7
Description: Gene set enrichment analysis of candidate genes

File Name: Supplementary Data 8
Description: List of 169 putative driver genes

File Name: Supplementary Data 9
Description: Gene set enrichment analysis of Notch1-driven TNBC tumors

File Name: Supplementary Data 10
Description: Sequence of oligos
