# Supplementary material for: NOTCH1 activation compensates BRCA1 deficiency and promotes triple-negative breast cancer formation
Source: Nat Commun. 2020 Jun 26;11:3256. doi: 10.1038/s41467-020-16936-9 (PMC7320176; doi:10.1038/s41467-020-16936-9)
Supplement: Supplementary file 14 — Reporting Summary [file 41467_2020_16936_MOESM14_ESM.pdf]

nature research

Corresponding author(s): Chu-Xia Deng

Last updated by author(s): May 15, 2020

Reporting Summary

Nature Research wishes to improve the reproducibility of the work that we publish. This form provides structure for consistency and transparency in reporting. For further information on Nature Research policies, see our Editorial Policies and the Editorial Policy Checklist.

Statistics

For all statistical analyses, confirm that the following items are present in the figure legend, table legend, main text, or Methods section.

n/a	Confirmed	
☐	☒	The exact sample size (n) for each experimental group/condition, given as a discrete number and unit of measurement
☐	☒	A statement on whether measurements were taken from distinct samples or whether the same sample was measured repeatedly
☐	☒	The statistical test(s) used AND whether they are one- or two-sided<br>*Only common tests should be described solely by name; describe more complex techniques in the Methods section.*
☒	☐	A description of all covariates tested
☒	☐	A description of any assumptions or corrections, such as tests of normality and adjustment for multiple comparisons
☐	☒	A full description of the statistical parameters including central tendency (e.g. means) or other basic estimates (e.g. regression coefficient) AND variation (e.g. standard deviation) or associated estimates of uncertainty (e.g. confidence intervals)
☐	☒	For null hypothesis testing, the test statistic (e.g. F, t, r) with confidence intervals, effect sizes, degrees of freedom and P value noted<br>*Give P values as exact values whenever suitable.*
☒	☐	For Bayesian analysis, information on the choice of priors and Markov chain Monte Carlo settings
☒	☐	For hierarchical and complex designs, identification of the appropriate level for tests and full reporting of outcomes
☒	☐	Estimates of effect sizes (e.g. Cohen's d, Pearson's r), indicating how they were calculated

Our web collection on statistics for biologists contains articles on many of the points above.

Software and code

Policy information about availability of computer code

Data collection	Human clinical databases TCGA and METABRIC were used for the NOTCH1 and TNBC correlation analysis. we used R studio (version 3.5.1) with "cgdsr" packages for the data download. Clinical pathology information was also retrieved.<br>All the other data supporting the findings of this study can be found within the supplementary files.
Data analysis	CISs identification were conducted with Bowtie2 version 2.3.4 followed by TAPDANCE analysis. The aligned locations identified as CISs were automatically annotated based on mouse reference genes (mm10.gtf).<br>RNA sequencing reads were aligned to the mouse reference genome mm10 and processed using HISAT2 version 2.1.0. Differential expression analysis was conducted using DESeq2 version 1.22.2.<br>TNBC correlation analysis was conducted by using R studio (version 3.5.1).<br>PAM50 subtype assignment was conducted using Genefu (2.18.1).<br>Gene function annotation enrichment analysis was performed with DAVID Bioinformatics v6.8 using the Gene Ontology and KEGG pathway datasets. GSEA (GSEA v3.0) was also used for gene expression difference analysis. Analysis of driver gene molecular interactions among CIS genes was conducted using STRING v11.0 online tools.

For manuscripts utilizing custom algorithms or software that are central to the research but not yet described in published literature, software must be made available to editors and reviewers. We strongly encourage code deposition in a community repository (e.g. GitHub). See the Nature Research guidelines for submitting code & software for further information.

Data

Policy information about availability of data

All manuscripts must include a data availability statement. This statement should provide the following information, where applicable:

- Accession codes, unique identifiers, or web links for publicly available datasets
- A list of figures that have associated raw data
- A description of any restrictions on data availability

The RNA sequence data have been deposited in the Sequence Read Archive (SRA) database under the accession code PRJNA529536 (https://www.ncbi.nlm.nih.gov/bioproject/PRJNA529536/). The data from TCGA and METABRIC referenced in the study are available in a public repository from the cBioPortal website (https://www.cbioportal.org/). All the other data supporting the findings of this study can be found within the supplementary files. A reporting summary for this article is available as a Supplementary Information file.

Field-specific reporting

Please select the one below that is the best fit for your research. If you are not sure, read the appropriate sections before making your selection.

☒ Life sciences ☐ Behavioural & social sciences ☐ Ecological, evolutionary & environmental sciences

For a reference copy of the document with all sections, see nature.com/documents/nr-reporting-summary-flat.pdf

Life sciences study design

All studies must disclose on these points even when the disclosure is negative.

Sample size	Tumor sample size were determined by quantity of tumor sample collection. Mouse sample cohort size were estimated based on the published literature. Based on the reported cases, the SB mice cohort usually are less than 100 mice, here we enlarged the animal number to 188 and 129 individually, which should be enough for tumor driver genes identification.<br>For cell line drug treatment experiment, we used 5 TNBC cell lines for the test which is sufficient for statistical analysis.
Data exclusions	No data were excluded.
Replication	All experiments were conducted at least three times independently, and similar results were adopted for further analysis to guarantee reproducibility.
Randomization	Mice used for tumor incidence analysis in Fig. 1b were allocated based on the genotype.<br>Mice used for drug treatment experiment which demonstrated in Fig. 8d were allocated randomly into each treatment groups.<br>Cells used for drug treatment experiments which listed in Fig. 8a, b, and c were allocated randomly into each treatment groups.
Blinding	Investigators were not blinded to mouse genotype regarding Figure 1. As we need to continually generate same genotype of the mice to reach the desired number of mice cohort, therefore the investigators were not blinded.<br>Investigators were blinded to the in vivo drug treatment experiments and other in vitro experiments which conducted on cell lines.<br>Investigators were not blinded to the Western blot experiments as people need to rank and load the samples based on the treatment information.

Reporting for specific materials, systems and methods

We require information from authors about some types of materials, experimental systems and methods used in many studies. Here, indicate whether each material, system or method listed is relevant to your study. If you are not sure if a list item applies to your research, read the appropriate section before selecting a response.

Materials & experimental systems

n/a	Involved in the study	
☐	☒	Antibodies
☐	☒	Eukaryotic cell lines
☒	☐	Palaeontology and archaeology
☐	☒	Animals and other organisms
☒	☐	Human research participants
☒	☐	Clinical data
☒	☐	Dual use research of concern

Methods

n/a	Involved in the study	
☒	☐	ChIP-seq
☐	☒	Flow cytometry
☒	☐	MRI-based neuroimaging

Antibodies

Antibodies used	IHC staining was performed with following antibodies: ER� (1:50, Santa Cruz, sc-542), PR (1:50, Santa Cruz, sc-538), HER2 (1:50, Santa

Antibodies used

Cruz, sc-284), cleaved caspase-3 (1:500, CST, 9664), and Ki67 (1:200, Abcam, ab16667). The secondary antibody were provided in the Histostain-Plus IHC kit (Thermo, 859043, Lot: 1954379A).
Antibodies used for western blotting were: BRCA1 (1:200, Santa Cruz, sc-642), NOTCH1 (1:1000, CST 4380), E-cadherin (1:1000, CST 3195), β-catenin (1:1000, Abcam, ab32572), N-cadherin (1:1000, Abcam, ab76057), Vimentin (1:1000, CST 5741), Fibronectin (1:1000, Abcam, ab2431), Slug (1:1000, CST 9585), β-actin (1:4000, Sigma, A5316), ATM (1:1000, Abcam ab78), pATM (1:1000, Abcam, ab81292), ATR (1:1000, CST 2790), pATR-1989 (1:1000, Abcam, ab227851), pATR-428 (1:1000, CST 2853), CHK1 (1:200, Santa Cruz, sc-56288), pCHK1-317 (1:1000,CST 12302), and pCHK1-345 (1:1000, CST 2348). The secondary antibodies used in this study were as follows: Anti-rabbit IgG, HRP-linked Antibody (1:5000, CST 7074, Lot:28), Anti-mouse IgG, HRP-linked Antibody (1:5000, CST 7076, Lot:32).
Antibodies for MI detection and Fluorescence microscopy were: p-H3 antibody (1:500, Millipore, 06-570), BrdU (1:200, Santa Cruz, sc-32323), γ-H2AX (1:500, Millipore, 05-636, Lot: 3076468) and 53BP1 (1:200, Santa Cruz, sc-22760, Lot: I1813). Secondary antibodies for this experiments are: F(ab')2-Goat anti-Rabbit IgG (H+L) Cross-Adsorbed Secondary Antibody, Alexa Fluor 488 (1:1000, Thermo, A-11070, Lot: 1494754), F(ab')2-Goat anti-Rabbit IgG (H+L) Cross-Adsorbed Secondary Antibody, Alexa Fluor 594 (1:1000, Thermo, A11072, Lot: 1431810), F(ab')2-Goat anti-Mouse IgG (H+L) Cross-Adsorbed Secondary Antibody, Alexa Fluor 594 (1:1000, Thermo, A-11020, Lot: 1454439), F(ab')2-Goat anti-Mouse IgG (H+L) Cross-Adsorbed Secondary Antibody, Alexa Fluor 488 (1:1000, Thermo, A-11017, Lot: 1557766)

Validation

Validation information was provided by manufacturer, published literature referenced in the manuscript.

Eukaryotic cell lines

Policy information about cell lines

Cell line source(s)

Sum149 cell line purchased from ExPASy, All other human cell lines were purchased from ATCC. Mouse primary mammary tumor cells were derived from SB tumors.

Authentication

STR testing.

Mycoplasma contamination

All cell lines were test negative for Mycoplasma contamination.

Commonly misidentified lines (See ICLAC register)

No commonly misidentified cell lines were used.

Animals and other organisms

Policy information about studies involving animals; ARRIVE guidelines recommended for reporting animal research

Laboratory animals

All mouse experiments were performed under the ethical guidelines of the University of Macau (animal protocol number: UMAEC-037-2015). The following mouse strains were used in this study: 1) Brca1 conditional knockout (Brca1Co/Co) mice, in which the deletion of exon 11 of Brca1 is controlled by two mammary tissue-specific Cre transgenes (WAP-Cre or MMTV-Cre). 2) Two strains with conditionally expressed SB11 transposase that includes a floxed transcriptional stop cassette to be activated by Cre. 3) and independent transgenic lines of T2onc3 (12740 and 12775). Due to several transgenic strains used in this study, the resulting cohorts of mice were on a mixed genetic background, including C57BL/6J, 129SVE, and FVB. Only female mice were used for the experiment, and all the mice were pregnant once at 2–4 months of age to activate the expression of WAP-Cre or MMTV-Cre.
The female Nude and NSG mice were used for tumor inoculation at 6 weeks old.
Mice were housed in a Specific-pathogen-free (SPF) facility at 23-25°C on a 12-h light/dark cycle.

Wild animals

No wild animals were used in the study.
Mice were housing in the SPF animal facility and monitored twice a week for tumorigenesis, and tumors were collected when they reached approximately 1–2 centimetres in diameter or the mice were moribund. Complete autopsy was performed to assess primary and metastatic tumors. Mouse was euthanasia with CO2 exposure.

Field-collected samples

No field collected samples were used in the study.

Ethics oversight

All mouse experiments were performed under the ethical guidelines of the University of Macau (animal protocol number: UMAEC-037-2015).

Note that full information on the approval of the study protocol must also be provided in the manuscript.

Flow Cytometry

Plots

Confirm that:

☐ The axis labels state the marker and fluorochrome used (e.g. CD4-FITC).

☒ The axis scales are clearly visible. Include numbers along axes only for bottom left plot of group (a 'group' is an analysis of identical markers).

☒ All plots are contour plots with outliers or pseudocolor plots.

☒ A numerical value for number of cells or percentage (with statistics) is provided.

Methodology

Sample preparation	cells were trypsinized and fixed in 70% cold ethanol for at least 1 hour. The cells were permeabilized with 0.25% Triton X-100 for 15 min at room temperature and incubated with 5% BSA/PBS to block the cells. Then, cells were incubated sequentially with p- H3 antibody (Millipore, 06-570), Alexa 488-conjugated secondary antibody and PI.
Instrument	BD FACSCALIBUR
Software	BD CellQuestTM Pro
Cell population abundance	The cell population abundance information were provided in the Source Data file.
Gating strategy	PI signaling reflect the DNA content in the cell. signal of Alexa 488 indicate p-H3 positive or negative.

☒ Tick this box to confirm that a figure exemplifying the gating strategy is provided in the Supplementary Information.
